# Biophysical Modeling to Determine the Optimization of Left Ventricular Pacing Site and AV/VV Delays in the Acute and Chronic Phase of Cardiac Resynchronization Therapy

**DOI:** 10.1111/jce.13134

**Published:** 2017-01-14

**Authors:** ANGELA W. C. LEE, ANDREW CROZIER, EOIN R. HYDE, PABLO LAMATA, MICHAEL TRUONG, MANAV SOHAL, THOMAS JACKSON, JONATHAN M. BEHAR, SIMON CLARIDGE, ANOOP SHETTY, EVA SAMMUT, GERNOT PLANK, CHRISTOPHER ALDO RINALDI, STEVEN NIEDERER

**Affiliations:** ^1^ Division of Imaging Sciences and Biomedical Engineering King's College London London UK; ^2^ Institute of Biophysics Medical University of Graz Graz Austria; ^3^ Cardiovascular Department Guy's and St. Thomas’ NHS Foundation Trust London UK

**Keywords:** atrioventricular delay, cardiac resynchronization therapy, computer modeling/simulations, interventricular delay, left bundle branch block, left ventricular lead placement

## Abstract

**Background:**

Cardiac anatomy and function adapt in response to chronic cardiac resynchronization therapy (CRT). The effects of these changes on the optimal left ventricle (LV) lead location and timing delay settings have yet to be fully explored.

**Objective:**

To predict the effects of chronic CRT on the optimal LV lead location and device timing settings over time.

**Methods:**

Biophysical computational cardiac models were generated for 3 patients, immediately post‐implant (ACUTE) and after at least 6 months of CRT (CHRONIC). Optimal LV pacing area and device settings were predicted by pacing the ACUTE and CHRONIC models across the LV epicardium (49 sites each) with a range of 9 pacing settings and simulating the acute hemodynamic response (AHR) of the heart.

**Results:**

There were statistically significant differences between the distribution of the AHR in the ACUTE and CHRONIC models (P < 0.0005 in all cases). The site delivering the maximal AHR shifted location between the ACUTE and CHRONIC models but provided a negligible improvement (<2%). The majority of the acute optimal LV pacing regions (76–100%) and device settings (76–91%) remained optimal chronically.

**Conclusion:**

Optimization of the LV pacing location and device settings were important at the time of implant, with a reduced benefit over time, where the majority of the acute optimal LV pacing region and device settings remained optimal with chronic CRT.

## Introduction

Cardiac resynchronization therapy (CRT) is an effective treatment for drug refractory patients with dyssynchronous heart failure; however, 30% of patients fulfilling current CRT implant criteria do not respond.^1^ CRT nonresponse is multifactorial and reflects underlying patient substrate, implantation issues including left ventricular (LV) pacing site location, and postimplant programming with suboptimal atrioventricular delay (AVD) and/or interventricular delay (VVD) settings.^2^

Optimal LV pacing location strategies have been proposed to maximize cardiac function at the time of implant, where the ideal LV site has been identified as being at the site of the latest mechanical^3^ or electrical activation^4^ and away from the apex and scarred regions of the heart.^5^, ^6^ Optimizing AV/VVD, while holding the pacing location fixed, has shown improvements in the acute hemodynamic response (AHR) at the time of implant.^7^, ^8^

The ability of AV/VVD optimization to partially compensate for suboptimal LV lead placement demonstrates the interdependence of these device settings.^9^, ^10^ Despite this, routine optimization of the AVD/VVD settings concurrently with the LV lead location optimization is typically not performed. Currently, there are no clinical guidelines for optimizing AV/VVD settings. Therefore, a common strategy is to use the default device settings and optimize for the LV lead location at the time of implant and only optimizing the AV/VVD for nonresponders at a later stage.^2^, ^11^, ^12^

The optimal AV/VVD settings and LV stimulation site have significant interindividual variation. This interindividual variability may be compounded by significant intraindividual variation within a single patient over time due to changes in the cardiac anatomy and function caused by sustained CRT. Due to dynamic changes in cardiac physiology and anatomy, whether it be positive (in responders) or negative (in nonresponders), the optimal pacing location and device timings may change dynamically. Optimizing pacing location and timings as the heart remodels over time may therefore offer a strategy for maximizing CRT response.^13^, ^14^ Dynamically optimizing timing delays during the course of CRT treatment remains controversial, with some studies showing improvement,^15^, ^16^ while others finding no improvements in clinical outcome with optimization after 3–6 months of CRT.^7^, ^17^ Recent advances in multisite LV stimulation from multipolar leads in a single vein or multivein pacing now offer the potential to noninvasively and dynamically optimize both the timing and location of pacing following device implantation to maximize patient response.^18^, ^19^ However, determining the best way to alter the pacing location and device timings remains a significant challenge.

Biophysical models of a patient's heart provide a framework for capturing patient physiology and pathology. Using models, the functional response of the heart can be simulated and thereby predict the optimal LV pacing location and the optimal AV/VVD timings. Modeling can be performed both to reflect the acute setting at implant and in the chronic setting following implantation after the heart has undergone LV reverse remodeling, with CRT associated changes in the geometry, electrophysiology,^20^, ^21^ loading conditions, and cardiac mechanics.^22^, ^23^

We have previously modeled the acute effects of CRT in relation to the optimal LV pacing location and validated these findings with AHR from actual patients.^24^, ^25^ The ability to create biophysical models to predict the optimal pacing site with chronic CRT following remodeling and the effects of AVD and VVD optimization is possible but has not previously been described, as this requires invasive data both at the time of implant and chronically following CRT, which is not routinely acquired in clinical practice. To perform this analysis, we used biophysical modeling in a small number of patients who had uniquely undergone invasive electrical and hemodynamic measures both at implant and after at least 6 months following CRT. We hypothesized that as the heart remodeled with chronic CRT, the optimal LV pacing location and AV/VVD combination and the benefits of optimizing these parameters would change over time.

## Methods

Data were acquired from 3 patients with standard indications for CRT, who had undergone both acute and chronic invasive hemodynamic pacing studies as part of dedicated research protocols.^26^ Patients were male, aged 64 ± 6 years with ischemic heart disease, 2‐dimensional (2D) ejection fraction was 20 ± 12%, and all patients had left bundle branch block on the surface electrocardiogram (ECG) with a mean QRS duration (QRSd) of 151 ± 25 ms, with New York Heart Association (NYHA) class III symptoms at the time of implant. In all cases, the LV lead was placed in the lateral or postero‐lateral branches of the coronary sinus vein using a multipolar LV lead (Quartet St. Jude Medical), while the right ventricle (RV) lead was placed in the RV apex.

For each patient, the CRT clinical response (NYHA class, Minnesota Living with Heart Failure Questionnaire score, and 6‐minute walk distance) and the LV reverse remodeling response (percentage change in 2D cardiac echo LV end systolic volume [ESV]) were recorded within 3 months prior to implant and after at least 6 months of CRT for the 3 patients, which is summarized in Table 1. In contrast to the improvement in the LV response for cases 1 and 2, an improvement in the clinical measures was reported for cases 2 and 3 (Table 1). In a previous work by Yu *et al*., it was found that though there was a tendency for an improvement in the clinical response to be correlated with long‐term survival, this was not statistically significant.^27^ A decrease of ≥10% LV ESV was found to be a strong predictor of the long‐term survival and lower heart failure events for patients,^27^ and has been used to classify the 3 patient cases into responders (cases 1 and 2) and nonresponders (case 3) in this study.

**Table 1:** Clinical Response (2D Echo End Systolic Volume [ESV] Change; New York Heart Association Functional Classification [NYHA Class]; Minnesota Living With Heart Failure Questionnaire [HF] Score; and 6‐Minute Walk Distance) for the 3 Patient Cases Within 3 Months Prior to Device Implantation (ACUTE) and After at Least 6 Months of Sustained Pacing (CHRONIC) Were Used to Classify Patients as Either Responders or Nonresponders to Cardiac Resynchronization Therapy (CRT)

Case	Time	ESV Change	NYHA Class	HF Score	6‐Minute Walk Distance	Responder?
Case 1	ACUTE	−67%	III	45	Unchanged	Y
	CHRONIC		III	55		
Case 2	ACUTE	−11%	III	36	280 m	Y
	CHRONIC		I	26	370 m	
Case 3	ACUTE	+36%	III	64	505 m	N
	CHRONIC		I	4	546 m	

All patients provided written informed consent for both procedures that were approved by the local research ethics committee. Patients underwent an acute hemodynamic pacing study at the time of CRT implant with a pressure wire (Radi wire, Radi Medical Systems, Uppsala, Sweden) placed into the LV cavity to measure the maximal change in pressure over time (dP/dt_max_) with atrial and biventricular (BiV) pacing. The relative improvement of dP/dt_max_ between atrial and BiV pacing was used to calculate the AHR of the patient to pacing (Table 2):

AHR = max  dP  dt  BiV − max  dP  dt  atrial  max  dP  dt  atrial

**Table 2:** The Clinical Functional Response of the Patient to Baseline (AAI) and Biventricular (DDD‐BiV) Pacing was Recorded at Time of Implant (ACUTE) and After at Least 6 Months of Sustained Pacing (CHRONIC)

Case	Time	Clinical AHR	Simulated AHR
Case 1	ACUTE	34.2%	30.1%
	CHRONIC	18.9%	18.1%
Case 2	ACUTE	17.8%	16.1%
	CHRONIC	19.2%	19.7%
Case 3	ACUTE	0.3%	6.1%
	CHRONIC	23.0%	20.5%

The pacing lead locations were mapped onto personalized biophysically based models of the heart for 3 patients. The heart models were used to simulate AAI and DDD‐BiV pacing at the ACUTE and CHRONIC points. The acute hemodynamic response (AHR) of the heart to pacing was calculated as the relative change in the maximal change in left ventricular pressure over time between DDD‐BiV and AAI pacing. The simulated AHR shows good agreement with the clinical AHR at both the ACUTE and CHRONIC time points.

All patients underwent magnetic resonance imaging (MRI) within 3 months prior to device implantation as a part of standard clinical practice. Segmentations of the 3‐dimensional (3D) whole heart and contrast‐enhanced MR images were used to personalize the cardiac anatomy and scar regions in the models prior to sustained pacing (ACUTE models).^24^, ^25^ The changes in the cardiac geometry after chronic CRT were represented in the models by deforming the ACUTE models to match segmentations from 2D and 3D echocardiography performed after sustained CRT (CHRONIC models) (see Supplementary Material).

After at least 6 months of CRT, a further invasive study was performed with pressure measurements and electro‐anatomical mapping of the LV cavity, acquired using a pressure wire and a noncontact mapping array (EnSite Array Catheter, St. Jude Medical, Minnetonka, MN, USA) placed in the LV, respectively.^26^ X‐ray fluoroscopy images were acquired during the invasive studies to track the location of the pacing leads. X‐ray MR image registration was used to determine the pacing locations in the model simulations.^28^

In this study, the models used to investigate CRT focused on the isovolumetric contraction, systole, and isovolumetric relaxation phases of the cardiac cycle; to simulate the phases of the cardiac cycle of interest; and to reduce computational costs. Large deformation mechanics were simulated using Continuum Mechanics, Image analysis, Signal processing and System Identification (CMISS) software (www.cmiss.org).

**Figure 1:**
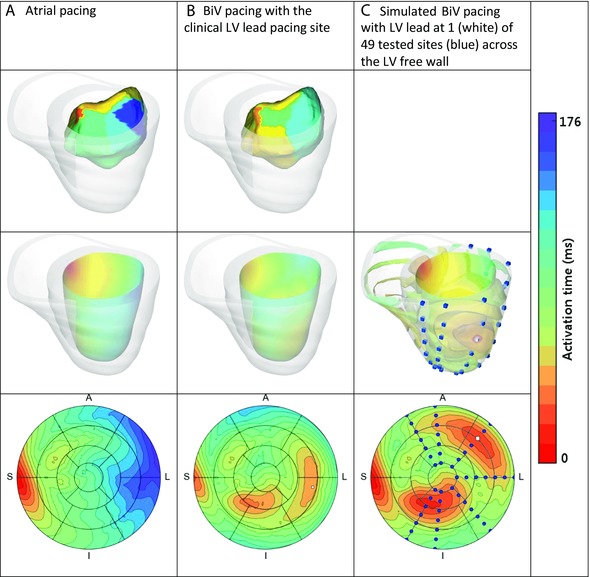
Top row: Clinical measurements of the LV endocardium electrical activation were taken using an ENSITE balloon catheter for case 2 with (A) atrial pacing and (B) biventricular (BiV) pacing. Middle row: The model parameters were fitted to the electrical activation of the heart with (A) atrial or (B) BiV pacing. (C) The models were then used to simulate the electrical activation of the heart at a range of LV epicardium sites. Bottom row: The simulated activation wave on the LV endocardium was projected onto the 17‐segment AHA map for the different scenarios.

The fiber architecture was set to vary from apex to base and transmurally using a previously described, rule‐based method derived from cadaveric and canine data^24^ (Supplementary Table S1). The electrophysiology of the heart was simulated using the Cardiac Arrhythmia Research Package.^29^ The electrical activation of the heart was simulated by pacing the heart at the intrinsic or paced activation sites and fitting the conductivity parameters to the QRSd determined from 12‐lead ECG. The geometry of the LV endocardium in the model at the CHRONIC stage was derived from 3D cardiac echo images, while the EnSite system maps the LV endocardial surface separately (Fig. 1). The model simulations of the electrical activation were qualitatively compared against the clinical measurements, as currently there is no standard method for mapping between the LV endocardial surfaces derived from medical images and the Ensite system. To quantitatively validate the model, we predicted activation times for additional protocols (RV or LV only pacing) and it was found that the electrical conductivity parameters led to an error between the model predicted and clinical QRSd of 7.1 ± 3.1 ms (see Supplementary Table S2).

The passive and active mechanics parameters and the pressure boundary condition model parameters were fitted based on the pressure‐volume curve (PV loop). The volume transients at the ACUTE and CHRONIC time points were acquired from semiautomatic tracking of the LV endocardial surface using TomTec software (www.tomtec.de) with ECG gated cine MR and 3D cardiac echo images, respectively. The volume transients were normalized with regard to the end diastolic volume of the LV. Volume transients were registered to the invasive pressure measurements using simultaneously recorded ECG to generate PV loops at ACUTE and CHRONIC time points, as described previously.^25^

**Figure 2:**
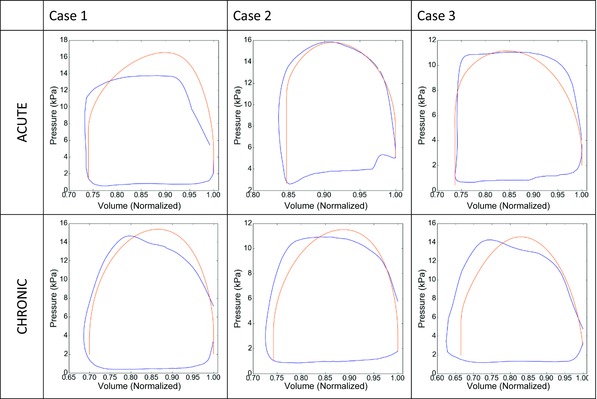
Clinical (blue) and simulated (orange) pressure volume loops for the 3 patient cases before (ACUTE) and after at least 6 months of CRT treatment (CHRONIC).

**Figure 3:**
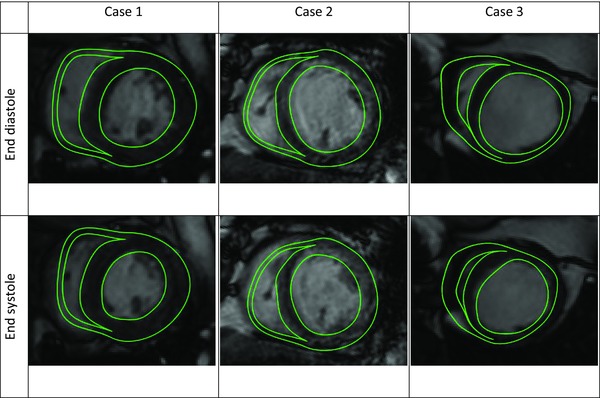
The motion of the cardiac models during the cardiac cycle was validated against cine MR images.

A 3‐element Windkessel model was used to represent the systemic blood flow and pressure out of the ventricles. The Windkessel and passive mechanic parameters in each model were fitted to the PV loops for each patient at ACUTE and CHRONIC time points.^25^ The active contraction of the heart was represented using a length‐dependent model.^24^ The active parameters were fitted to both the PV loops and the AHR of the heart with atrial and BiV pacing for the ACUTE and CHRONIC models for the 3 cases (Fig. 2). The function of the heart, as reproduced with the AHR for the ACUTE and CHRONIC studies, shows a good agreement between the measured and modeled AHR (Table 2). Epicardial and endocardial wall motion contours are overlaid in the cine MRI to validate the simulated motion of the heart throughout the modeled phases of the heart cycle as shown in Figure 3.

### Quantification of the Size of the Optimal Pacing Region

Pacing sites across the LV free wall were simulated using the models to determine the relative roles of LV pacing location and timing settings on AHR with chronic CRT over time. Actual LV coronary sinus pacing is limited to sites accessible *via* the coronary branch anatomy, whereas in computer models activation can be simulated at all points across the myocardium. In each ACUTE and CHRONIC model, 49 simulations were run, where the LV pacing site was varied to span the LV epicardium (Fig. 1). Each LV pacing site was activated along with the RV lead pacing site and the intrinsic activation sites, for 9 AV/VVD permutations. The AHR was evaluated across the LV epicardium and interpolated to provide a continuous estimate of AHR at all locations. The optimal pacing region was defined as the area within 70% of the global maximal AHR as proposed previously by Helm *et al*.^30^ and was plotted on the AHA model.

### AV/VVD Optimization Simulation

The modeling framework focuses on the ventricles and does not explicitly represent the atria. The AVD is the time delay between when atrial pacing occurs to when the ventricles are paced. The intrinsic electrical activation of the heart from the atria passes through the atrio‐ventricular node, where it then travels via the fast conducting Purkinje network to activate the ventricles. The effect of changing AVD was approximated by changing the timing of the intrinsic activation that is set by atrial pacing, and the delay to the ventricle pacing leads. A short AVD where the ventricle leads are activated early is modeled by the absence of any intrinsic activation. A fused AVD is modeled by simultaneous activation of the ventricle leads and intrinsic activation, while a long AVD delay is modeled by ventricle lead activation 40 ms after the intrinsic activation. Three VVDs were modeled with –20, 0, and +20 ms with positive values for early RV pacing.^31^ This gives 9 AVD/VVD permutations.

The ACUTE and CHRONIC models were paced across the LV free wall (49 sites) with 9 different AV/VVD combinations giving 441 simulations per model (2,646 simulations in total). In contrast to a sequential optimization, where the LV pacing location, AVD, and then VVD timing delays are successively optimized, the exhaustive search method that we have taken calculates the global maximal AHR for each case for the ACUTE and CHRONIC models across all locations and timing combinations.

### Statistics

For each case, ACUTE and CHRONIC models were used to simulate pacing at 9 device settings and 49 locations across the LV free wall to predict the AHR. Values are reported as the mean ± standard deviation. Post‐hoc comparisons of the 441 simulations of the AHR between the ACUTE and CHRONIC time points were made using paired *t*‐tests for each patient case. P < 0.05 was considered statistically significant.

## Results

Biophysically based models of the heart were generated for the 3 patient cases at both the ACUTE and CHRONIC time points. Simulations of pacing at 49 different sites across the LV epicardium with 9 different AV/VVD device settings were used to predict the AHR of the heart to pacing at the ACUTE and CHRONIC time points.

### Difference Between ACUTE and CHRONIC Models

To determine if the optimal LV pacing location and the AV/VVD settings change with chronic CRT, in each patient case, a paired *t*‐test was used to compare the AHR distribution over the LV free wall between the ACUTE and CHRONIC models. The mean ± SD for the AHR across the LV free wall for the 9 timing variations for the ACUTE and CHRONIC models was: case 1: 32.9 ± 18.2% and 18.0 ± 7.7%; case 2: 4.4 ± 5.7% and 5.9 ± 7.9%; and case 3: 2.1 ± 1.7% and 5.7 ± 5.8%, respectively, with P < 0.0005 in all cases.

### Benefits of LV Lead Location Optimization

**Figure 4:**
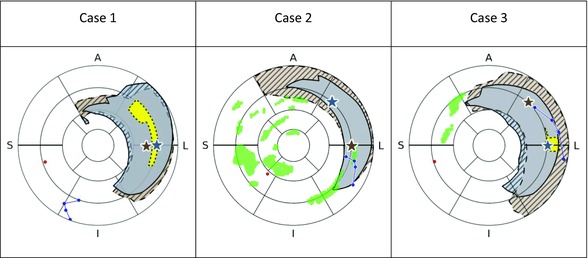
The area where any 1 of the 9 combinations of AV/VVD timings would give an optimal response is shown (ACUTE: gray with solid outline; CHRONIC: brown with dashed outline). Areas of no overlap between the optimal regions for CHRONIC and ACUTE models are shown as striped. Regions in the CHRONIC models where all 9 of the AV/VVD combinations would give an optimal response are shown as yellow (dotted outline) for cases 1 and 3. There were no such regions in the ACUTE model and the CHRONIC model for case 2. The locations of the multipolar LV lead (blue), RV lead (red), and scar (green) are shown for all cases. The location of the maximal response for any of the 9 AV/VVD timings is shown as stars (ACUTE: gray; CHRONIC: brown).

Figure 4 shows the optimal area and maximal AHR site for LV pacing for the ACUTE and CHRONIC models. Across all 3 cases, the majority of the optimal region from the ACUTE model remained within the CHRONIC optimal region. In case 2, the optimal pacing region in the ACUTE model laid entirely within that of the CHRONIC model. For cases 1 and 3, the optimal pacing area shifted, predicting that if the pacing lead was within the ACUTE optimal region, then it had an 82% and 76% probability, respectively, of remaining optimal after chronic CRT. The best single site for pacing the LV shifted in all cases after sustained pacing (Fig. 4); however, altering the pacing site from the maximal ACUTE to the maximal CHRONIC pacing location only improved the AHR by <2% for all cases (case 1: 0.4%, case 2: 1.3%, and case 3: 1.2%).

### Benefits of Timing Delay Optimization

**Figure 5:**
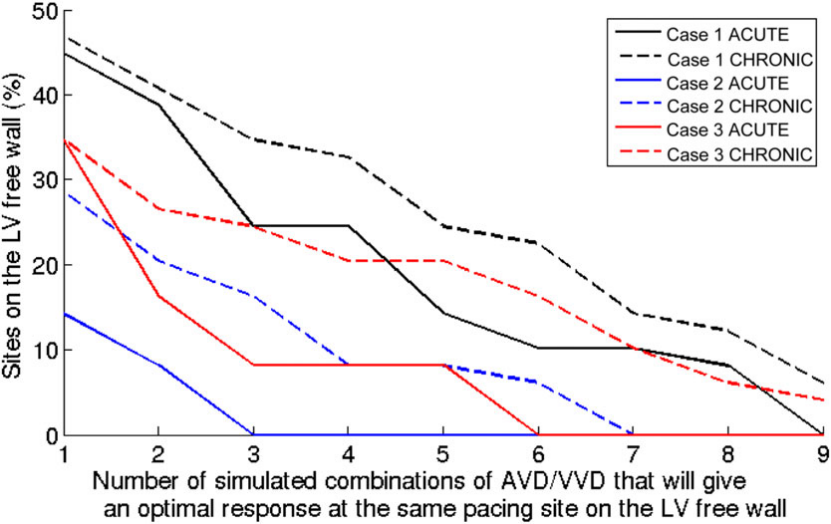
The LV free wall was paced and the AHR was simulated at 49 locations with 9 AV/VVD settings in the ACUTE and CHRONIC time points for each case. The optimal LV pacing regions were defined as the LV sites within 70% of the global maximal AHR. The percentages of the 49 sites on the LV free wall that gives an optimal response for pacing with 1–9 of the simulated AV/VVD timing settings for the ACUTE and CHRONIC models are shown for the 3 cases.

Figure 5 shows the percentage of the 49 evaluated pacing locations that achieve an optimal response for different numbers of combinations of AV/VVD timings. In all 3 case studies, the predicted number of AV/VVD/pacing locations that delivered optimal AHR increased between the ACUTE and CHRONIC CRT models (20–26% for case 1; 3–10% for case 2; and 8–18% for case 3). The majority of optimal AV/VVD settings in the ACUTE models are predicted to remain optimal chronically (case 1: 88%, case 2: 91%, and case 3: 76%) at the same LV pacing site.

## Discussion

ACUTE and CHRONIC biophysical models were generated for 3 case studies to predict the relative importance of LV lead location and AV/VVD optimization in the acute response of the heart immediately post‐implant and after sustained CRT treatment.

Our results are the first to model both acute and chronic response to CRT in humans and show that:

(1) The model replicates measured hemodynamic and electrical indices for both the ACUTE and CHRONIC time points.
(2) Lead optimization and AV/VVD optimization at the time of implant were important in improving acute and chronic response.
(3) The models predicted that the benefits of AV/VVD and lead location optimization decreased over time with sustained pacing, i.e., with chronic CRT, there was less incremental benefit seen with AV/VVD or lead position optimization.

### Benefits of Optimizing the LV Lead Location Over Time

Acute optimization of the LV lead location is a recognized strategy for maximizing CRT response.^3^, ^4^, ^5^, ^6^ Optimizing the pacing location acutely using multisite pacing has shown improved AHR^19^, ^32^; however, the ability to dynamically optimize pacing location post‐implant with these new lead technologies has yet to be evaluated. At both acute and chronic time points, our models predicted distinct pacing regions that gave optimal AHR that covered most of the LV lateral free wall, consistent with acute animal studies,^30^ clinical studies,^33^ and clinical guidelines for CRT.^11^ Our models predicted that the majority (76–100%) of the ACUTE optimal pacing locations remain optimal in the CHRONIC case. The maximal AHR site shifted for all 3 cases as the heart remodeled (Fig. 4) supporting postimplant lead location optimization; however, the predicted improvement in AHR achieved by moving from the best ACUTE to the best CHRONIC pacing location in the CHRONIC model was small (<2%). The model indicates that it is more important to optimize the LV lead location at the time of implant, that this location is likely to be in the LV lateral wall, and that in the majority of locations this site will remain optimal as the heart remodels.

### Benefits of Optimizing the AV/VVD Over Time

Studies have shown the acute benefits of optimizing the AV/VVD^7^, ^9^ and our model simulations also reflected this, where optimizing the AV/VVD settings could improve the AHR (dependent on the default settings) at the ACUTE time point (Fig. 5). The slope and the area under the curve for the ACUTE traces in Figure 5 reflect how beneficial optimizing between the simulated AV/VVD combinations would be. The greater the slope of the curve and the smaller the area under the curve is, the less overlap there is between the optimal areas of LV pacing for any of the 9 simulated combinations. In the chronic setting, we find that as the heart remodels, the number of timing combinations that deliver an optimal response increases (shown as a rightward shift of all curves in Fig. 5), consistent with previous studies that have not found a consistent benefit of dynamic AV/VVD optimization.^7^, ^17^ In addition, our models predicted that an optimal AV/VVD/LV pacing site will likely remain optimal after sustained pacing indicating that it is more important to optimize for the AV/VVD at the time of implant.

### CRT Nonresponder

In this study, response/nonresponse was classified based on an improvement of the LV response (≥10% decrease in the LV ESV^27^). Case 3 was classed as a nonresponder based on the LV response deteriorating (LV ESV +36%). This is in keeping with the poor AHR acutely measured (0.3%) and predicted by the model (6.1%). However, after sustained pacing, the measured AHR increased (from 0.3% ACUTE to 23.0% CHRONIC) (Table 2). An increase in the AHR in the nonresponder seems counter‐intuitive; however, if we consider that if the LV response of the heart continues to deteriorate for case 3, then there is greater capacity for improvement with BiV pacing. In addition, we have shown that the models predicted that the site of maximal AHR shifts after sustained pacing (Fig. 4). The predicted maximal AHR site in the ACUTE model was further from the LV pacing lead than in the CHRONIC model (ACUTE: 33 mm; CHRONIC model: 15 mm). The improvement in the proximity of the LV pacing lead to the ideal pacing site, in combination with the increased capacity of response to pacing, could contribute to the observed increase of the AHR for this case.

## Limitations

In this study, we have only used 3 clinical case studies at 2 time points to generate our results; thus, the conclusions drawn need to be taken in this context. The extensive clinical data required to build and validate biophysical models of patient's hearts limit the expansion of this framework to large groups. We have chosen instead to run an in‐depth study for these 3 patients, where 2,646 simulations of different pacing locations and timing delays were run to explore the relative importance of LV lead and AV/VVD optimization over time.

The range of AVD and VVD tested was limited to only 3 settings each, giving 9 combinations of device timing settings to be run over 49 different sites per model (441 simulations), to remain computationally tractable. Instead of optimizing for AVD at increments of 20 ms over a wide range of AVD values as performed clinically,^9^, ^15^ we chose to represent 3 scenarios for the AVD: a short AVD, a fused AVD, and a long AVD. The VVD was set to be –20, 0, and 20 ms, as previous work found that the optimal VVD lies between ± 20 ms.^31^

The model accounted for scar at the time of implant, but we assumed the location, degree, and properties of the scar at the ACUTE time remained constant in the CHRONIC model. Nor did we account for phrenic nerve stimulation. Changes in either of these conditions at a local pacing site could require the pacing location to be altered post‐implant, which could be enabled by multisite pacing lead technologies.

In this study, we optimized the LV pacing location and the AV/VVD settings using the AHR as a measure of the function of the heart. However, the link between the AHR and LV reverse remodeling remains controversial, with some studies finding no link,^34^ while others have found a positive correlation between AHR and clinical outcome.^35^ Other measures of acute improvement such as diastolic parameters and pressure volume loop have also been proposed; however, they have yet to be linked to long‐term clinical response.^1^, ^22^

## Clinical Relevance

The heart anatomy and physiology dynamically remodels in response to CRT. We have generated biophysical patient‐specific models to predict how these changes affect the optimal CRT lead pacing location and device timings. We find that there is diminished benefit in postimplant optimization of the pacing location or timings as the heart reverse remodels with the majority of settings remaining optimal as the heart responds to CRT. This emphasizes the importance of ensuring good lead position and optimized device settings at the time of implant.

## Conclusion

In this study, patient‐specific biophysical models were used as a framework to predict the optimal LV pacing location and the effects of AV/VVD optimization immediately post‐implant and after at least 6 months of CRT for 3 case studies. The models predict that although the optimal pacing region can shift, the majority of the optimal pacing sites remain in the LV lateral wall. The models predict that optimization of lead position and AV/VVD optimization are more beneficial at the time of implant than after sustained CRT pacing.

## Supporting information


**Table S1**. The fiber orientations in the models were defined using a rule‐based method that varies the fiber and sheet angles of between the right ventricle (RV) and left ventricle (LV), through the endocardium (Endo) to the epicardium (epi), and from apex to base. The septum is between the right and left ventricles and thus there is no epicardium surface to be defined.
**Table S2**. The electrophysiology of the heart was simulated for different pacing protocols: Atrial, Biventricular (BiV), Right ventricle (RV), and Left ventricle (LV). The QRS duration for the ACUTE and CHRONIC models was calculated as the time it takes for the ventricles to be electrically activated. This was compared against the clinical QRSd measured from ECGs.

## References

[1] Chung ES , Leon AR , Tavazzi L , Sun J‐P , Nihoyannopoulos P , Merlino J , Abraham WT , Ghio S , Leclercq C , Bax JJ , Yu C‐M , Gorcsan J , St John Sutton M , De Sutter J , Murillo J : Results of the predictors of response to CRT (PROSPECT) trial. Circulation 2008;117:2608‐2616. 18458170 10.1161/CIRCULATIONAHA.107.743120

[2] Mullens W , Grimm RA , Verga T , Dresing T , Starling RC , Wilkoff BL , Tang WHW : Insights from a cardiac resynchronization optimization clinic as part of a heart failure disease management program. J Am Coll Cardiol 2009;53:765‐773. 19245967 10.1016/j.jacc.2008.11.024

[3] Ypenburg C , van Bommel RJ , Delgado V , Mollema SA , Bleeker GB , Boersma E , Schalij MJ , Bax JJ : Optimal left ventricular lead position predicts reverse remodeling and survival after cardiac resynchronization therapy. J Am Coll Cardiol 2008;52:1402‐1409. 18940531 10.1016/j.jacc.2008.06.046

[4] Zanon F , Baracca E , Pastore G , Fraccaro C , Roncon L , Aggio S , Noventa F , Mazza A , Prinzen F : Determination of the longest intrapatient left ventricular electrical delay may predict acute hemodynamic improvement in patients after cardiac resynchronization therapy. Circ Arrhythm Electrophysiol 2014;7:377‐383. 24668162 10.1161/CIRCEP.113.000850

[5] Khan FZ , Virdee MS , Fynn SP , Dutka DP : Left ventricular lead placement in cardiac resynchronization therapy: Where and how? Europace 2009;11:554‐561. 19372115 10.1093/europace/eup076

[6] Singh JP , Klein HU , Huang DT , Reek S , Kuniss M , Quesada A , Barsheshet A , Cannom D , Goldenberg I , McNitt S , Daubert JP , Zareba W , Moss AJ : Left ventricular lead position and clinical outcome in the Multicenter Automatic Defibrillator Implantation Trial‐Cardiac Resynchronization Therapy (MADIT‐CRT) trial. Circulation 2011;123:1159‐1166. 21382893 10.1161/CIRCULATIONAHA.110.000646

[7] Boriani G , Biffi M , Müller CP , Seidl KH , Grove R , Vogt J , Danschel W , Schuchert A , Deharo JC , Becker T , Boulogne E , Trappe HJ : A prospective randomized evaluation of VV delay optimization in CRT‐D recipients: Echocardiographic observations from the RHYTHM II ICD study. Pacing Clin Electrophysiol 2009;32:S120‐S125. 19250074 10.1111/j.1540-8159.2008.02267.x

[8] Bogaard MD , Meine M , Tuinenburg AE , Maskara B , Loh P , Doevendans PA : Cardiac resynchronization therapy beyond nominal settings: Who needs individual programming of the atrioventricular and interventricular delay? Europace 2012;14:1746‐1753. 22753868 10.1093/europace/eus170

[9] Khan FZ , Virdee MS , Read PA , Pugh PJ , Begley D , Fynn SP , Dutka DP : Impact of VV optimization in relation to left ventricular lead position: An acute haemodynamic study. Europace 2011;13:845‐852. 21427090 10.1093/europace/eur037 PMC3101849

[10] Lane RE , Chow AW , Mayet J , Francis DP , Peters NS , Schilling RJ , Davies DW : The interaction of interventricular pacing intervals and left ventricular lead position during temporary biventricular pacing evaluated by tissue Doppler imaging. Heart 2007;93:1426‐1432. 17277351 10.1136/hrt.2006.087445 PMC2016892

[11] Vardas PE , Auricchio A , Blanc J‐J , Daubert J‐C , Drexler H , Ector H , Gasparini M , Linde C , Morgado FB , Oto A , Sutton R , Trusz‐Gluza M: Guidelines for cardiac pacing and cardiac resynchronization therapy. The Task Force for Cardiac Pacing and Cardiac Resynchronization Therapy of the European Society of Cardiology. Developed in collaboration with the European Heart Rhythm Association. Europace 2007;9:959‐998. 17726043 10.1093/europace/eum189

[12] Cuoco FA , Gold MR : Optimization of cardiac resynchronization therapy: Importance of programmed parameters. J Cardiovasc Electrophysiol 2012;23:110‐118. 22188487 10.1111/j.1540-8167.2011.02235.x

[13] Linde C , Gold MR , Abraham WT , St John Sutton M , Ghio S , Cerkvenik J , Daubert C : Long‐term impact of cardiac resynchronization therapy in mild heart failure: 5‐year results from the REsynchronization reVErses Remodeling in Systolic left vEntricular dysfunction (REVERSE) study. Eur Heart J 2013;34:2592‐2599. 23641006 10.1093/eurheartj/eht160

[14] Punn R , Hanisch D , Motonaga KS , Rosenthal DN , Ceresnak SR , Dubin AM : A pilot study assessing ECG versus ECHO ventriculoventricular optimization in pediatric resynchronization patients. J Cardiovasc Electrophysiol 2016;27:210‐216. 26515428 10.1111/jce.12863

[15] Sawhney NS , Waggoner AD , Garhwal S , Chawla MK , Osborn J , Faddis MN : Randomized prospective trial of atrioventricular delay programming for cardiac resynchronization therapy. Heart Rhythm 2004;1:562‐567. 15851220 10.1016/j.hrthm.2004.07.006

[16] Abraham WT , León AR , St. John Sutton MG, Keteyian SJ, Fieberg AM, Chinchoy E, Haas G: Randomized controlled trial comparing simultaneous versus optimized sequential interventricular stimulation during cardiac resynchronization therapy. Am Heart J 2012;164:735‐741. 23137504 10.1016/j.ahj.2012.07.026

[17] Ellenbogen KA , Gold MR , Meyer TE , Fernández Lozano I , Mittal S , Waggoner AD , Lemke B , Singh JP , Spinale FG , Van Eyk JE , Whitehill J , Weiner S , Bedi M , Rapkin J , Stein KM : Primary results from the SmartDelay determined AV optimization: A comparison to other AV delay methods used in cardiac resynchronization therapy (SMART‐AV) trial. A randomized trial comparing empirical, echocardiography‐guided, and algorithmic atrioventricular delay programming in cardiac resynchronization therapy. Circulation 2010;122:2660‐2668. 21098426 10.1161/CIRCULATIONAHA.110.992552

[18] Rinaldi CA , Kranig W , Leclercq C , Kacet S , Betts T , Bordachar P , Gutleben K , Keel A , Ryu K , Farazi TG , Simon M , Naqvi T : Multisite left ventricular pacing improves acute mechanical dyssynchrony in heart failure patients. J Am Coll Cardiol 2012;59:E918. 10.1016/j.cardfail.2013.10.003 24263116

[19] Zanon F , Baracca E , Pastore G , Marcantoni L , Fraccaro C , Lanza D , Picariello C , Aggio S , Roncon L , Dell'Avvocata F , Rigatelli G , Pacetta D , Noventa F , Prinzen FW : Multipoint pacing by a left ventricular quadripolar lead improves the acute hemodynamic response to CRT compared with conventional biventricular pacing at any site. Heart Rhythm 2015;12:975‐981. 25625721 10.1016/j.hrthm.2015.01.034

[20] Aiba T , Hesketh GG , Barth AS , Liu T , Daya S , Chakir K , Dimaano VL , Abraham TP , O'Rourke B , Akar FG , Kass DA , Tomaselli GF : Electrophysiological consequences of dyssynchronous heart failure and its restoration by resynchronization therapy. Circulation 2009;119:1220‐1230. 19237662 10.1161/CIRCULATIONAHA.108.794834 PMC2703676

[21] Sachse FB , Torres NS , Savio‐Galimberti E , Aiba T , Kass DA , Tomaselli GF , Bridge JH : Subcellular structures and function of myocytes impaired during heart failure are restored by cardiac resynchronization therapy. Circ Res 2012;110:588‐597. 22253411 10.1161/CIRCRESAHA.111.257428 PMC3299196

[22] Steendijk P , Tulner SA , Bax JJ , Oemrawsingh PV , Bleeker GB , van Erven L , Putter H , Verwey HF , van der Wall EE , Schalij MJ : Hemodynamic effects of long‐term cardiac resynchronization therapy: Analysis by pressure‐volume loops. Circulation 2006;113:1295‐1304. 16520415 10.1161/CIRCULATIONAHA.105.540435

[23] Søgaard P , Egeblad H , Kim WY , Jensen HK , Pedersen AK , Kristensen BØ , Mortensen PT : Tissue Doppler imaging predicts improved systolic performance and reversed left ventricular remodeling during long‐term cardiac resynchronization therapy. J Am Coll Cardiol 2002;40:723‐730. 12204503 10.1016/s0735-1097(02)02010-7

[24] Niederer SA , Plank G , Chinchapatnam P , Ginks M , Lamata P , Rhode KS , Rinaldi CA , Razavi R , Smith NP : Length‐dependent tension in the failing heart and the efficacy of cardiac resynchronization therapy. Cardiovasc Res 2011;89:336‐343. 20952413 10.1093/cvr/cvq318

[25] Crozier A , Blazevic B , Lamata P , Plank G , Ginks M , Duckett S , Sohal M , Shetty A , Rinaldi CA , Razavi R , Smith NP , Niederer SA : The relative role of patient physiology and device optimisation in cardiac resynchronisation therapy: A computational modelling study. J Mol Cell Cardiol 2015;96:93‐100. 26546827 10.1016/j.yjmcc.2015.10.026 PMC4915816

[26] Shetty AK , Sohal M , Chen Z , Ginks MR , Bostock J , Amraoui S , Ryu K , Rosenberg SP , Niederer SA , Gill J , Carr‐White G , Razavi R , Rinaldi CA : A comparison of left ventricular endocardial, multisite, and multipolar epicardial cardiac resynchronization: An acute haemodynamic and electroanatomical study. Europace 2014;16:873‐879. 24525553 10.1093/europace/eut420

[27] Yu C‐M , Bleeker GB , Fung JW‐H , Schalij MJ , Zhang Q , van der Wall EE , Chan Y‐S , Kong S‐L , Bax JJ : Left ventricular reverse remodeling but not clinical improvement predicts long‐term survival after cardiac resynchronization therapy. Circulation 2005;112:1580‐1586. 16144994 10.1161/CIRCULATIONAHA.105.538272

[28] Truong M , Gordon T , Razavi R , Penney G , Rhode K : Analysis of catheter‐based registration with vessel‐radius weighting of 3D CT data to 2D X‐ray for cardiac catheterisation procedures in a Phantom study In: Camara O, Konukoglu E, Pop M, Rhode K, Sermesant M, Young A (eds.): Statistical Atlases and Computational Models of the Heart Imaging and Modelling Challenges. Berlin, Heidelberg: Springer, 2012;7085:139‐148.

[29] Niederer S , Mitchell L , Smith N , Plank G : Simulating human cardiac electrophysiology on clinical time‐scales. Front Physiol 2011;2:14. 21516246 10.3389/fphys.2011.00014 PMC3079856

[30] Helm RH , Byrne M , Helm PA , Daya SK , Osman NF , Tunin R , Halperin HR , Berger RD , Kass DA , Lardo AC : Three‐dimensional mapping of optimal left ventricular pacing site for cardiac resynchronization. Circulation 2007;115:953‐961. 17296857 10.1161/CIRCULATIONAHA.106.643718

[31] Søgaard P , Egeblad H , Pedersen AK , Kim WY , Kristensen BØ , Hansen PS , Mortensen PT : Sequential versus simultaneous biventricular resynchronization for severe heart failure: Evaluation by tissue Doppler imaging. Circulation 2002;106:2078‐2084. 12379577 10.1161/01.cir.0000034512.90874.8e

[32] Rinaldi CA , Leclercq C , Kranig W , Kacet S , Betts T , Bordachar P , Gutleben K‐J , Shetty A , Donal E , Keel A , Ryu K , Farazi TG , Simon M , Naqvi TZ : Improvement in acute contractility and hemodynamics with multipoint pacing via a left ventricular quadripolar pacing lead. J Interv Card Electrophysiol 2014;40:75‐80. 24626999 10.1007/s10840-014-9891-1

[33] Butter C , Auricchio A , Stellbrink C , Fleck E , Ding J , Yu Y , Huvelle E , Spinelli J : Effect of resynchronization therapy stimulation site on the systolic function of heart failure patients. Circulation 2001;104:3026‐2029. 11748094 10.1161/hc5001.102229

[34] Suzuki H , Shimano M , Yoshida Y , Inden Y , Muramatsu T , Tsuji Y , Tsuboi N , Hirayama H , Shibata R , Murohara T : Maximum derivative of left ventricular pressure predicts cardiac mortality after cardiac resynchronization therapy. Clin Cardiol 2010;33:E18‐E23. 21184541 10.1002/clc.20683 PMC6653395

[35] Duckett SG , Ginks M , Shetty AK , Bostock J , Gill JS , Hamid S , Kapetanakis S , Cunliffe E , Razavi R , Carr‐White G , Rinaldi CA : Invasive acute hemodynamic response to guide left ventricular lead implantation predicts chronic remodeling in patients undergoing cardiac resynchronization therapy. J Am Coll Cardiol 2011;58:1128‐1136. 21884950 10.1016/j.jacc.2011.04.042

