# Supplementary material for: Biophysical Modeling to Determine the Optimization of Left Ventricular Pacing Site and AV/VV Delays in the Acute and Chronic Phase of Cardiac Resynchronization Therapy
Source: J Cardiovasc Electrophysiol. 2017 Jan 14;28(2):208–15. doi: 10.1111/jce.13134 (PMC5535003; doi:10.1111/jce.13134)
Supplement: Supplementary file 1 — Table S1. The fiber orientations in the models were defined using a rule‐based method that varies the fiber and sheet angles of between the right ventricle (RV) and left ventricle (LV), through the endocardium (Endo) to the epicardium (epi), and from apex to base. The septum is between the right and left ventricles and thus there is no epicardium surface to be defined. Table S2. The electrophysiology of the heart was simulated for different pacing protocols: Atrial, Biventricular (BiV), Right ventricle (RV), and Left ventricle (LV). The QRS duration for the ACUTE and CHRONIC models was calculated as the time it takes for the ventricles to be electrically activated. This was compared against the clinical QRSd measured from ECGs. [file JCE-28-208-s001.docx]

**SUPPLEMENTAL MATERIAL**

Further details of the clinical data acquisition, model personalization, and validation are given in this supplement.

The study aimed to investigate the differences in the relative importance of the pacing location and device settings as the heart remodels over time with cardiac resynchronization therapy (CRT). Biophysically based computer models of the heart were used to simulate the contraction of the heart when it was paced at different locations across the LV epicardium and with varied device settings. In contrast to previous work (where a full description of the modeling framework is given) ^1, 2^, in this study two models of the heart were developed for each patient, representing the state of the heart both before and after CRT remodeling.

**Clinical data:**

Clinical data from 3 patients were acquired at two different time points: prior to/immediately post implant of the CRT device (ACUTE) and after at least 6 months of sustained CRT (CHRONIC) with fully informed written consent as per protocols approved by the local ethics committee. The models were personalized to each patient, with the pressure boundary conditions, geometry, mechanics, and electrical activation parameters fitted and validated from clinical data at the ACUTE and CHRONIC time points.

Within 3 months prior to CRT implant (ACUTE), MR images were acquired using a 1.5T Philips Achieva system and steady-state free procession (SSFP) images were acquired of the 3D whole heart at the end diastolic phase of the cardiac cycle (0.8 x 0.8 x 1mm). 2D short axis cine SSFP (1.4x1.4x10mm, 30 cardiac phases) and Gadolinium late-enhancement (1.25x1.25x10mm) breath-hold scans were also acquired.

At initial implant (ACUTE), bilateral femoral access was used to place pacing catheters in the heart at the right ventricular (RV) apex, high right atria and left ventricle (LV). The LV was paced via the coronary sinus with a quadripolar catheter (Quartet St Jude Medical). The LV endocardial pressure changes were measured with a high fidelity wire pressure wire (Radi wire, Radi Medical Systems, Uppsala, Sweden) in the LV cavity. The heart was paced with the following protocol: at the high right atria for the baseline setting, RV-only, LV-only, or with simultaneous biventricular pacing (BiV) at 5-10 beats above the intrinsic heart rate with AVD 100ms and VVD 0ms where appropriate. The heart was paced at the high right atria, in between each of the other paced protocols and the pressure in the LV was recorded 20s after any change in pacing setting to ensure hemodynamic stability. As part of standard clinical practice, X-ray fluoroscopy images of the heart were acquired to track the location of the catheters during the procedure.

After at least 6 months of sustained pacing (CHRONIC), pressure measurements were repeated with the same pacing protocol to determine the effects of sustained pacing. A non-contact mapping array (EnSite Array Catheter, St Jude Medical, Minnetonka, MN, USA) was also passed into the LV cavity to reconstruct the LV endocardial potential. As above, X-ray fluoroscopy images were also acquired and the invasive electroanatomical maps were aligned to the MRI data using the XMR registration technique developed by Rhode *et al. ^3^.* In addition 12 lead ECGs, real time 3D echocardiograms and 2-chamber, 3-chamber, and 4-chamber 2D cardiac echos were acquired at both time points.

**Geometric fitting:**

The geometry of the heart has been found to change with CRT treatment either positively (reverse remodeling) or negatively (continued deterioration with non-response to treatment)^4^. To personalize the geometry of the models to each patient, meshes were fit to medical images of the heart acquired from 3 patients before (ACUTE models) and after sustained CRT (CHRONIC models).

*ACUTE geometry*

Cardiac MR images were acquired as a part of standard clinical practice. SSFP MR images of the 3D whole heart at end diastole were segmented using Philips Model Based Segmentation Framework to generate binary masks of the ventricles^5^. Cubic Hermite meshes (144 elements, 228 or 231 nodes, C^1^ continuous) were then fitted to the ventricle segmentations for each patient (ACUTE models) with a mean RMS error of <1mm^6^. Late Gadolinium enhancement MR images were used to identify regions of myocardial infarction, which were then mapped to the ACUTE models.

*Fiber structure*

Tissue microstructure orientation was described using a rule based fiber distribution motivated by canine^7-9^ and human^10^ studies that varied transmurally, in the apex-base direction, and between the left and right ventricle as shown below in Table 1:

Table 1. The fiber orientations in the models were defined using a rule-based method that varies the fiber and sheet angles of between the right ventricle (RV) and left ventricle (LV), through the endocardium (Endo) to the epicardium (epi), and from apex to base. The septum is between the right and left ventricles and thus there is no epicardium surface to be defined.

| Region | Sheet angle | | | Fiber angle | | |
| --- | --- | --- | --- | --- | --- | --- |
|  | Endo | Mid | Epi | Endo | Mid | Epi |
| LV free wall base | 0° | 30° | -40° | 60° | 0° | -60° |
| LV free wall apex | -20° | -20° | 15° | 83° | 24° | -35° |
| LV septum base | 0° | 0 | n/a | 60° | 0 | n/a |
| LV septum apex | 20° | 10 | n/a | 83° | 11.5 | n/a |
| RV free wall base | 0° | -20 | -40° | 60° | 0 | -60° |
| RV free wall apex | -20° | -2.5 | 15° | 60° | -12.5 | -35° |
| RV septum base | 0° | 0 | n/a | -60° | 0 | n/a |
| RV septum apex | 0° | 10 | n/a | -60° | 11.5 | n/a |

*CHRONIC geometry*

Due to the implantation of the CRT device, MRI was not performed on the patient post-implant and instead 2D and 3D cardiac echo images were used to define the geometry of the heart after sustained CRT. Real time 3D cardiac echo images of the LV endocardium at end diastole were segmented using TomTec software ([www.tomtec.de](http://www.tomtec.de)). The LV epicardium was not consistently captured in the 3D cardiac echo images, so 2D cardiac echo images (2-chamber, 3-chamber, and 4-chamber) were used to provide an estimate of the thickness of the LV. The biventricular ACUTE mesh was then warped to fit the new dimensions of the LV. The cardiac echo window similarly did not consistently capture the RV; therefore, in the absence of clinical data describing the change in the RV with sustained treatment, the RV wall thickness and RV cavity were assumed to be unchanged from the ACUTE time point. The scar and fiber orientation in the CHRONIC models were mapped from the ACUTE meshes ^1, 2^.

**Mechanics fitting:**

The heart deforms substantially throughout the cardiac cycle and to simulate the large deformation mechanics Continuum Mechanics, Image analysis, Signal processing and System Identification (CMISS) software ([www.cmiss.org](http://www.cmiss.org)) was used.

The passive and active mechanics parameters and the pressure boundary condition model parameters were fitted based on the pressure-volume curve (PV loop). Invasive pressure data was acquired at both the ACUTE and CHRONIC time points as previously described in Shetty et. al. ^11^. Short axis SSFP cine MR images were acquired at the ACUTE time point. Volume transients at the ACUTE time point were obtained from semi-automatic segmentations and tracking of the LV endocardium in the cine MR images by clinical experts using TomTec software. After CRT implant (CHRONIC), patients can no longer be imaged with MRI scanners, and therefore the volumetric transient of the left ventricle after sustained pacing were obtained from the segmentation and tracking of the LV endocardium throughout the cardiac cycle in 3D cardiac echo images by clinical experts using TomTec software. The volume was normalized to the end diastolic volume of the LV at each time point. The normalized volume and pressure data were then synchronized using the ECG recorded during the pressure or volume data acquisition to generate a PV loop ^1^, which was then used to constrain the model parameters.

*Passive mechanics*

As described in previous work ^1, 2^, passive material properties of viable cardiac tissue were modeled using a transversely isotropic hyperelastic constitutive law ^12^:

$W=C_{1}e^{\alpha Q}$ (1)

$Q={E_{ff}}^{2}+0.5\left( {E_{ss}}^{2}+{E_{nn}}^{2}+{{2E}_{ns}}^{2} \right)+0.5\left( {E_{fs}}^{2}+{E_{nf}}^{2} \right)$, (2)

where *E_ff_, E_ss_* and *E_nn_* are the Green strain aligned with the fiber, sheet and sheet normal orientations, *E_ns_*, *E_fn_* and *E_fs_* are the corresponding Green shear strains. The passive material properties of the ventricles, *C_1_* and *α* were fit to the PV loop during atrial contraction when the ventricles were assumed to be quiescent.

Scarred regions in the heart were assumed to be isotropic which required a reformulation of Eq.2 so that:

$Q={E_{ff}}^{2}+{E_{ss}}^{2}+{E_{nn}}^{2}+{E_{ns}}^{2}+{E_{fs}}^{2}+{E_{nf}}^{2}$ (3)

A five-fold increase in α and doubled *C_1_*, with respect to the viable tissue, was used to approximate the increased stiffness due to the higher density of collagen in scarred tissue as observed in animal studies^13^.

*Pressure boundary conditions*

The pressure boundary conditions representing the systemic circulation were simulated with a 3-element Windkessel model, where the change in flow is given by ^2^:

$\frac{dU}{dt}=\left( \frac{1}{CZ}+\frac{1}{RC} \right)U-\frac{P}{CZR}-\frac{1}{Z}\frac{dP}{dt}$, (4)

where U is the flow rate, C and R are the systemic compliance and resistance, respectively, Z is the aortic resistance and P is the LV pressure. The values for C, R and Z in the right ventricle were set to 4.5, 0.125 and 0.35 times the left ventricle values based on prior experimental data ^14-16^.

*Active mechanics*

A phenomenological length dependent active tension model, based a simplification of previous work done by Kerckoffs et al.^17^ was used to represent the active material properties of the model ^2^:

$\phi=tanh\left( a_{6}\left( \lambda-a_{7} \right) \right)$ (5)

$t_{r}=t_{r0}+a_{4}\left( 1-\phi\right)$ (6)

$T_{a}=\left\{ \begin{matrix} T_{0}\phi{tanh}^{2}{\left( \frac{t}{t_{r}} \right)tanh}^{2}\left( \frac{t_{max}-t}{t_{d}} \right) & & 0<t<t_{max} \\ 0 & & else \end{matrix} \right.$ (7)

where a_6_ corresponds to the length degree of length dependence, a_7_ corresponds to the length where no active tension is generated, a_4_ is the scalar of length dependent activation, t_r0_ is the baseline activation time constant, t_d_ is the relaxation time constant, t_max_ is the duration of tension generation, λ is the stretch in the fiber direction, T_a_ is the active tension, t_r_ regulates the rise time of the tension transit, φ is the nonlinear length dependent function and T_o_ is the peak isometric tension. The model of active tension was fitted to the isovolumetric contraction, systolic, and isovolumetric relaxation phases of the PV loop and the acute hemodynamic response (AHR) through successive parameter sweeps with Latin hypercube sampling.

**Electrophysiology fitting**

The electrophysiology of the heart was modeled using monodomain simplification of the bidomain equations:

$C_{m}\frac{dV_{m}}{dt}+I_{ion}=\nabla\cdot D_{m}\nabla V_{m}$ (8)

where *C_m_* is the membrane capacitance, *V_m_* is the transmembrane potential, *I_ion_* is transmembrane current, and *D_m_* is the transversely isotropic conductivity tensor. The transmembrane current (*I_ion_*) was simulated using a detailed biophysically-based human cell model by ten Tussher^18^. The electrophysiology of the mesh was then solved using the Cardiac Arrhythmia Research Package (CARP)^19^, on high resolution, irregular, linear tetrahedral meshes (19-44 million vertices, 145-260 million elements, mean edge length 250µm), which were derived from the cubic Hermite ACUTE and CHRONIC geometry meshes. The associated fiber and scarred regions from the cubic Hermite meshes were mapped to the electrophysiology meshes ^1, 2^.

*ACUTE electrophysiology*

To model the spread of electrical activation across the electrophysiology meshes, the location of the stimulation sites were defined. X-ray fluoroscopy images acquired during the CRT device implantation were registered against MR segmentation of the heart to identify the locations of the pacing leads ^20^, which were then mapped onto the ACUTE and CHRONIC models.

To fit the conductivity in the electrophysiology models, ECG data was recorded at both time points and the recorded QRS duration (QRSd) was taken to represent the time taken for the electrical activation to spread across both ventricles. In all cases, the QRSd data was available for atrial pacing at the ACUTE time point. Atrial pacing was simulated in the models and the ACUTE model conductivities were fit using the measured QRSd for atrial pacing.

*CHRONIC electrophysiology*

The location of the RV and LV intrinsic activation sites were based on the activation maps recorded by Durrer et. al.^21^ and EnSite electro-anatomical maps at the CHRONIC time point ^2^, respectively. In the absence of this data at the ACUTE time point, it was assumed that the intrinsic activation sites would remain at the same relative locations in the mesh, so the intrinsic activation sites from the CHRONIC meshes were mapped onto the ACUTE meshes.

At the CHRONIC time point, QRSd data was available for cases 2 and 3, when the heart was paced with atrial, LV only, RV only and BiV pacing. For case 1, only BiV pacing QRSd data was measured. The conductivity of the ACUTE and CHRONIC models were fitted using one pacing protocol (atrial pacing where possible or with BiV pacing). The simulated spread of electrical activation across the LV endocardium in the model was qualitatively compared against the endocardial calculated Ensite map^1^. A qualitative comparison was carried out as the Ensite system derives the endocardial surface separate from the MR images as shown in Figure 1 and mapping between these two geometries would inevitably introduce errors in the fit. The electrophysiology of the models were quantitatively validated using clinical data acquired when the heart was paced with different settings at the CHRONIC time point (BiV, RV, LV) for cases 2 and 3. The mean error for the QRSd between the models and clinical data for cases 2 and 3 was 7.1 ± 3.1ms in the CHRONIC stage (Table 2).

(A)
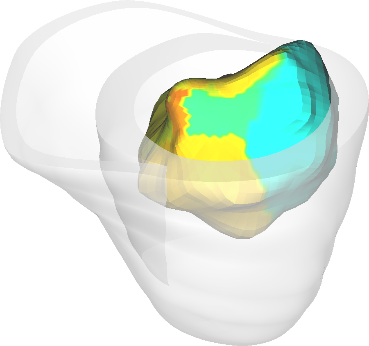
 (B)
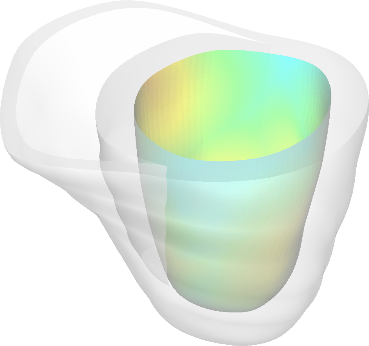


Figure 1. The spread of electrical activation across the LV endocardium surface from (A) the Ensite system and (B) derived from MRI data were qualitatively compared due to the discrepancy between the geometries.

Table 2. The electrophysiology of the heart was simulated for different pacing protocols: Atrial, Biventricular (BiV), Right ventricle (RV), and Left ventricle (LV). The QRS duration for the ACUTE and CHRONIC models was calculated as the time it takes for the ventricles to be electrically activated. This was compared against the clinical QRSd measured from ECGs.

| Case | time | pacing | Simulated QRSd (ms) | Clinical QRSd(ms) |
| --- | --- | --- | --- | --- |
| Case 1 | ACUTE | Atrial | 151.5 | 152 |
|  | ACUTE | BiV | 112.1 | * |
|  | CHRONIC | Atrial | 180.5 | * |
|  | CHRONIC | BiV | 141.4 | 141 |
|  | CHRONIC | RV | 166.6 | * |
|  | CHRONIC | LV | 166.8 | * |
| Case 2 | ACUTE | Atrial | 176.0 | 176 |
|  | ACUTE | BiV | 149.1 | * |
|  | CHRONIC | Atrial | 190.4 | 190 |
|  | CHRONIC | BiV | 151.7 | 157 |
|  | CHRONIC | RV | 190.2 | 183 |
|  | CHRONIC | LV | 151.9 | 162 |
| Case 3 | ACUTE | Atrial | 126.2 | 126 |
|  | ACUTE | BiV | 89.8 | * |
|  | CHRONIC | Atrial | 188.2 | 188 |
|  | CHRONIC | BiV | 138.8 | 130 |
|  | CHRONIC | RV | 179.8 | 178 |
|  | CHRONIC | LV | 214.5 | 205 |

** Clinical data not available*

**Model Limitations**

The biomechanical models of the heart were created based on a considerable and detailed clinical data; however, there was still insufficient information to fully characterize the models, therefore assumptions and simplifications had to be made.

The microstructure of the cardiac tissue is an important factor in how the electrical activation spreads throughout the heart and the mechanical functioning of the heart. Common methods for determining the fiber orientation of the cardiac tissue have been through histology or diffusion tensor (DT) MRI. The majority of DT MRI cardiac studies have been performed on ex vivo hearts^22, 23^, though recently studies have been performed in vivo with healthy human subjects^24, 25^. Lengthy acquisition times for DT MRI, low resolution and motion artifacts from heart motion and breathing are still challenges that need to be overcome before this method can be applied to clinical cases. In the absence of information regarding the orientation of the fibers in the tissue for the individual patient cases in this study, we have approximated the fiber orientation in the heart model by using a generic fiber model based on histological studies of canine and human data^10^. Earlier studies have shown similarities between the myofiber orientation between human and canine data^26^ and that the mechanics and electrophysiology models are robust to errors in minor perturbations of the fiber orientations.

Another limitation of the models is that the regions of the heart were set to be either infarcted or normal based on contrast enhanced MRI. Within the regions classified as infarcted or normal, the mechanical and electrical model parameters were assumed to be homogeneous. However, regional heterogeneities have been found even across normal cardiac tissue, with the material properties varying across the heart transmurally, apical-basally, and between the ventricles and also across the septum, and it has been found that the degree of heterogeneity can increase within infarcted tissue. The heterogeneities within the viable and scar regions were not taken into account in the models as there is currently an incomplete understanding of how the material properties vary across the heart, particularly under pathological conditions. In addition, while it is possible within the model framework to simulate heterogeneous material properties across the model, the clinical data collected for the 3 patients was insufficient to accurately personalize heterogeneous parameters within the normal and infarct regions. Therefore, we have chosen to use a homogeneous simplification of the material properties within each region.

**References**

[1] Crozier A, Blazevic B, Lamata P, Plank G, Ginks M, Duckett S, Sohal M, Shetty A, Rinaldi CA, Razavi R, Smith NP, Niederer SA: The relative role of patient physiology and device optimisation in cardiac resynchronisation therapy: A computational modelling study. Journal of Molecular and Cellular Cardiology 2015; 96:93-100.

[2] Niederer SA, Plank G, Chinchapatnam P, Ginks M, Lamata P, Rhode KS, Rinaldi Ca, Razavi R, Smith NP: Length-dependent tension in the failing heart and the efficacy of cardiac resynchronization therapy. Cardiovascular research 2011; 89:336-343.

[3] Rhode KS, Sermesant M, Brogan D, Hegde S, Hipwell J, Lambiase P, Rosenthal E, Bucknall C, Qureshi SA, Gill JS, Razavi R, Hill DLG: A system for real-time XMR guided cardiovascular intervention. Medical Imaging, IEEE Transactions on 2005; 24:1428-1440.

[4] Sutton MSJ, Keane MG: Reverse remodelling in heart failure with cardiac resynchronisation therapy. Heart (British Cardiac Society) 2007; 93:167-171.

[5] Peters J, Ecabert O, Meyer C, Schramm H, Kneser R, Groth A, Weese J: *Automatic whole heart segmentation in static magnetic resonance image volumes*. Medical Image Computing and Computer-Assisted Intervention–MICCAI 2007: Springer, 2007, pp. 402-410.

[6] Lamata P, Niederer S, Barber D, Norsletten D, Lee J, Hose R, Smith N: Personalization of cubic Hermite meshes for efficient biomechanical simulations. Medical image computing and computer-assisted intervention : MICCAI International Conference on Medical Image Computing and Computer-Assisted Intervention 2010; 13:380-387.

[7] Takayama Y, Costa KD, Covell JW: Contribution of laminar myofiber architecture to load-dependent changes in mechanics of LV myocardium. American journal of physiology Heart and circulatory physiology 2002; 282:H1510-1520.

[8] Usyk TP, Mazhari R, McCulloch AD: Effect of Laminar Orthotropic Myofiber Architecture on Regional Stress and Strain in the Canine Left Ventricle. 2000:143-164.

[9] LeGrice IJ, Smaill BH, Chai LZ, Edgar SG, Gavin JB, Hunter PJ: Laminar structure of the heart: ventricular myocyte arrangement and connective tissue architecture in the dog. American Journal of Physiology - Heart and Circulatory Physiology 1995; 269:H571-H582.

[10] Greenbaum RA, Ho SY, Gibson DG, Becker AE, Anderson RH: Left ventricular fibre architecture in man. British heart journal 1981; 45:248-263.

[11] Shetty AK, Sohal M, Chen Z, Ginks MR, Bostock J, Amraoui S, Ryu K, Rosenberg SP, Niederer SA, Gill J, Carr-White G, Razavi R, Rinaldi CA: A comparison of left ventricular endocardial, multisite, and multipolar epicardial cardiac resynchronization: an acute haemodynamic and electroanatomical study. Europace 2014; 16:873-879.

[12] Omens JH, MacKenna DA, McCulloch AD: Measurement of strain and analysis of stress in resting rat left ventricular myocardium. Journal of Biomechanics 1993; 26:665-676.

[13] Jugdutt BI, Joljart MJ, Khan MI: Rate of Collagen Deposition During Healing and Ventricular Remodeling After Myocardial Infarction in Rat and Dog Models. Circulation 1996; 94:94-101.

[14] Karamanoglu M, Bennett T: A Right Ventricular Pressure Waveform Based Pulse Contour Cardiac Output Algorithm in Canines. Cardiovascular Engineering 2006; 6:83-92.

[15] Santamore WP, Burkhoff D: Hemodynamic consequences of ventricular interaction as assessed by model analysis. Am J Physiol Heart Circ Physiol 1991; 260:H146-157.

[16] Heerdt PM, Gandhi CD, Dickstein ML: Disparity of isoflurane effects on left and right ventricular afterload and hydraulic power generation in swine. Anesth Analg 1998; 87:511-521.

[17] Kerckhoffs RCP, Bovendeerd PHM, Prinzen FW, Smits K, Arts T: Intra- and interventricular asynchrony of electromechanics in the ventricularly paced heart. 2003:201-216.

[18] ten Tusscher KHWJ, Panfilov AV: Alternans and spiral breakup in a human ventricular tissue model. Am J Physiol Heart Circ Physiol 2006; 291:H1088-1100.

[19] Niederer S, Mitchell L, Smith N, Plank G: Simulating human cardiac electrophysiology on clinical time-scales. Frontiers in physiology 2011; 2:14.

[20] Truong MV, Aslam A, Rinaldi CA, Razavi R, Penney GP, Rhode K: *Preliminary investigation: 2D-3D registration of MR and X-ray cardiac images using catheter constraints*. CI2BM09-MICCAI Workshop on Cardiovascular Interventional Imaging and Biophysical Modelling, 2009, pp. 9 pages.

[21] Durrer D, van Dam RT, Freud GE, Janse MJ, Meijler FL, Arzbaecher RC: Total excitation of the isolated human heart. Circulation 1970; 41:899-912.

[22] Lombaert H, Peyrat JM, Croisille P, Rapacchi S, Fanton L, Cheriet F, Clarysse P, Magnin I, Delingette H, Ayache N: Human Atlas of the Cardiac Fiber Architecture: Study on a Healthy Population. IEEE Transactions on Medical Imaging 2012; 31:1436-1447.

[23] Rohmer D, Sitek A, Gullberg GT: Reconstruction and Visualization of Fiber and Laminar Structure in the Normal Human Heart from Ex Vivo Diffusion Tensor Magnetic Resonance Imaging (DTMRI) Data. Investigative Radiology 2007; 42:777-789.

[24] Toussaint N, Stoeck CT, Schaeffter T, Kozerke S, Sermesant M, Batchelor PG: In vivo human cardiac fibre architecture estimation using shape-based diffusion tensor processing. Medical Image Analysis 2013; 17:1243-1255.

[25] Nielles-Vallespin S, Mekkaoui C, Gatehouse P, Reese TG, Keegan J, Ferreira PF, Collins S, Speier P, Feiweier T, de Silva R, Jackowski MP, Pennell DJ, Sosnovik DE, Firmin D: In vivo diffusion tensor MRI of the human heart: Reproducibility of breath-hold and navigator-based approaches. Magnetic Resonance in Medicine 2013; 70:454-465.

[26] Peyrat JM, Sermesant M, Pennec X, Delingette H, Chenyang X, McVeigh ER, Ayache N: A Computational Framework for the Statistical Analysis of Cardiac Diffusion Tensors: Application to a Small Database of Canine Hearts. Medical Imaging, IEEE Transactions on 2007; 26:1500-1514.
